# A surgical approach in the treatment of preputial gland abscesses in mice

**DOI:** 10.1186/s12917-016-0636-5

**Published:** 2016-01-19

**Authors:** Henri G. M. J. Bertrand, Aurelie A. Thomas, Yvette C. Ellen, Robert S. Dorward, Paul A. Flecknell

**Affiliations:** Comparative Biology Centre, Newcastle University, Framlington Place, Newcastle Upon Tyne, NE2 4HH UK; School of Veterinary Medicine and Science, Nottingham University, Nottingham, UK; Institute of Neurosciences, Newcastle University, Newcastle Upon Tyne, UK

**Keywords:** Refinement, Preputial, Gland, Surgery, Abscess

## Abstract

**Background:**

Preputial gland infection is a common occurrence in non-breeder male mice and can lead to abscesses. This report describes a surgical approach to treating and preventing this condition.

**Results:**

Surgical removal of the glands was undertaken in 258 male C3H/HeNHsd mice. The glands were successfully removed in all of the animals with a low rate of post-surgery complications. Abscess recurrence due to incomplete gland resection occurred in 2.3 % of animals. Surgical wound opening (3.1 %) and infection of the surgical site (2.3 %) also occurred but were treated successfully.

**Conclusion:**

In the study described here, early intervention was successful in preventing intercurrent infection compromising both animal welfare and the outcome of the study.

## Background

**Fig. 1:**
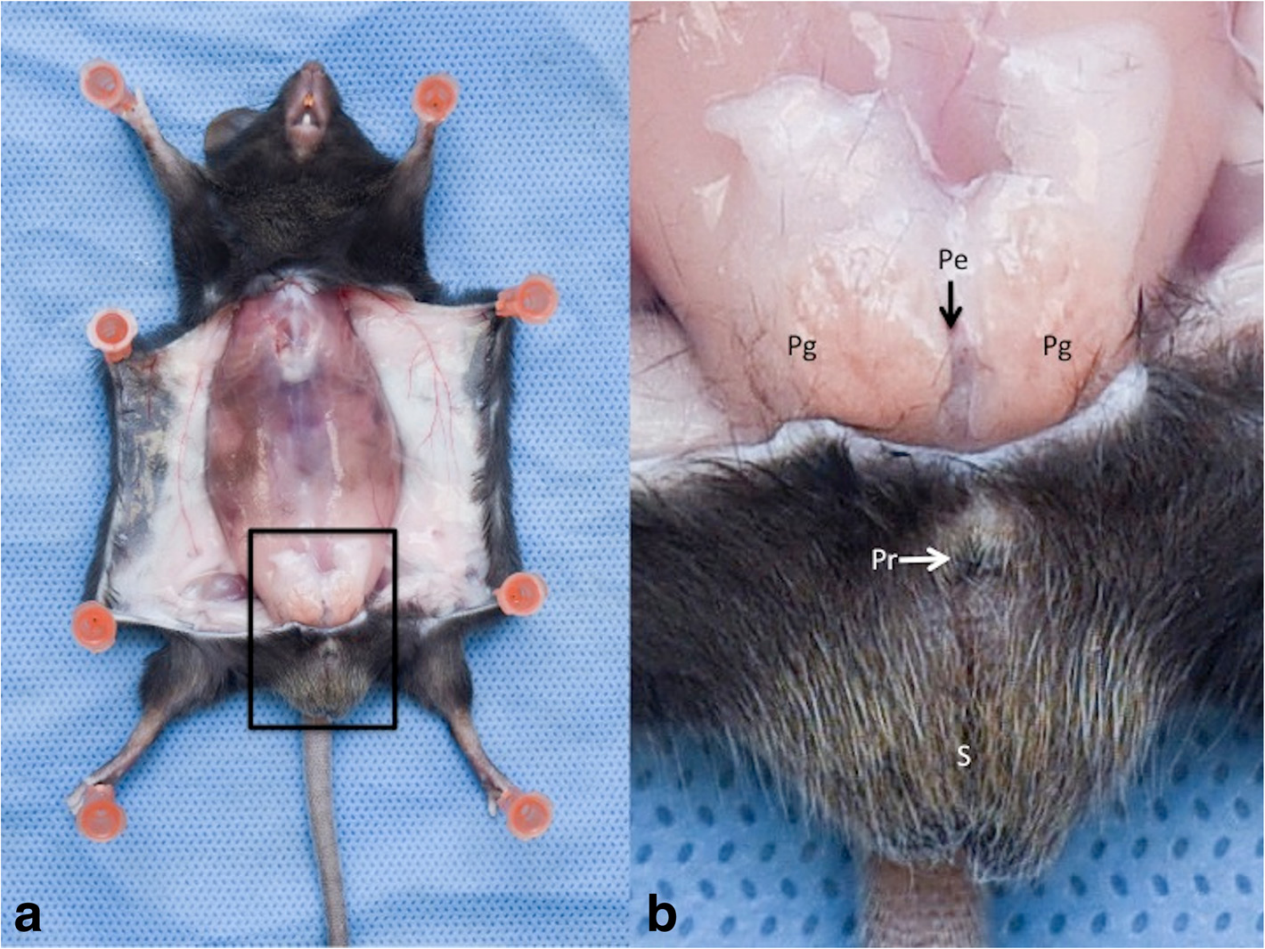
Anatomic Region of the preputial glands. **a** General view. The abdominal skin layer has been reclined to expose the muscle layer and the preputial glands region. The black frame represents the area enlarged in the next picture. **b** Enlarged view of the preputial glands region. (S = Scrotum; Pr = Prepuce; Pg = Preputial gland; Pe = Penis)

Preputial glands are accessory glands of the reproductive system of the male mouse and play a role in reproduction and dominance behavior [1, 2]. Inflammation and infection of these glands can lead to abscess formation. This health issue occurs more frequently in some mouse strains such as B6C3F1 [3, 4] however the incidence is usually quite low in C3H mice [5, 6]. The usual presentation of preputial gland inflammation is a uni- or bilateral subcutaneous swelling on either side of the prepuce. This develops to form a palpably hard mass and the overlying skin can be inflamed. In the worst case the abscesses can ulcerate and purulent exudate can be visible on the coat (Fig. 1). During a long-term (12 month) study in our facility, a high incidence of abscesses were noted, and the principal investigator was concerned at the impact of chronic infection and antibiotic treatment on both mouse welfare and the scientific goals of the study. To try to resolve the problem, a procedure to remove the glands surgically was developed, and this was used both to treat established abscesses, and as a preventive measure.

## Results

No complications related to the anaesthesia protocol occurred. In a small number of animals minor bleeding from the fatty tissue around the gland occurred but this was easily managed by applying pressure on the tissue with a dry swab. Given the location, perforation of the abdominal muscle layer or trauma to the penis could occur, but in our cases none of these complications occurred. The most common complication was re-opening of the surgical wound. Eight mice (3.1 %) removed skin sutures within 24 h post surgery, and the wound was closed again under general anaesthesia. Infection of the surgical wound, with obvious exudate occurred in six mice (2.3 %) within 24–48 h post surgery. Antibiotics were administered until infection resolved (typically 72 h). Recurrence of an abscess at the surgical site was noted in six mice (2.3 %).

## Discussion

The incidence of preputial gland inflammation or abscess are influenced by several factors. Mouse strain, age and microbiological flora are considered to be of importance [3–10] but also environmental condition such as group housing have been considered to increase the incidence of the condition [2]. In this case report, the high prevalence of this condition in C3H/HeNHsd mice was considered to be due to the high fat diet and the sugar water regimen used for the main study but no bacterial culture was performed to identify an infectious agent, as this would not have impacted our treatment approach. No histopathology exam was performed to confirm the diagnosis of abscess. This diagnosis was only based on the clinical signs observed and the similar progression of clinical signs in all animals. The treatment suggested in the literature is to lance the abscess and administer antibiotics to the animals [1, 7, 11]. However, in our experience the recurrence rate was high (95 %) when using this treatment regimen. This might be due to the multilobular structure of the gland preventing complete drainage of the abscess. The recurrence of these abscesses therefore represents a welfare cost for mice, as it may be associated with pain, fever and can lead to systemic amyloidosis [10]. Both a surgical procedure and repeated antibiotic therapy can impact on the welfare of the mice [12, 13]. The surgical procedure described has the advantages of preventing infection, and resolving established abscesses. It was associated with a low level of complication (in total 20 mice, 7.7 %). However this procedure is more technically demanding and time consuming than the previously documented treatment regimen. Like other surgical procedures, principles of good aseptic technique should be followed and a non-sterile assistant should be available to prepare the animal for surgery, monitor the anaesthesia and assist the surgeon. The time taken to perform each individual surgery was not recorded but in our experience each surgery required just under 10 min. Isoflurane was considered to be the anaesthetic of choice for this procedure as it provides a fast and smooth induction and recovery and also reliably produces a surgical plane of anaesthesia [14]. The other issue with this procedure, which was not investigated, is the potential impact on reproductive function since these glands play a role during the mating [1]. As a consequence, we only advise surgical removal of the preputial glands if a high incidence of the condition appears associated with a specific study, and when other means of reducing the incidence of the condition have been considered.

## Conclusion

In the study described here, early intervention was successful in preventing intercurrent infection compromising both animal welfare and the outcome of the study.

## Methods

### Ethical statement

Animals were housed in a Home Office (UK authority) accredited facility and in compliance with the Animal Scientific Procedure Act 1986 and the European Directive 2010/63/EU. Newcastle University Animal Welfare and Ethic Review Body and the Home Office approved research protocols. The procedure was performed under the United Kingdom Veterinary Surgeon’s Act 1969 with the agreement of the principal investigator and the Home Office.

### Animals and husbandry

This report is based on treatment of 258 male mice C3H/HeNHsd (from Harlan Laboratories, UK) involved in a study of the effect of several diets on the formation of liver fibrosis. Animals were aged from 6 to 10 weeks at the start of the study and the first signs of gland inflammation were noted between 18 and 20 weeks of age. In total, 56 mice (21.7 %) developed at least one gland abscess. The mice were housed in Individually Ventilated Cages (580 cm^2^, 226 mm × 419 mm × 147 mm, Arrowmight, UK), with 20 air cycles per hour. The day-night cycle was 12–12 h and the temperature was maintained at 22+/− 2 °C. During the study, an increasing number of mice presented with inflammation of their preputial glands. Because of the large number of affected animals, and because medical management of similar cases had proved unsuccessful, surgical removal of the gland was proposed.

### Surgical procedure and perioperative cares

**Fig. 2:**
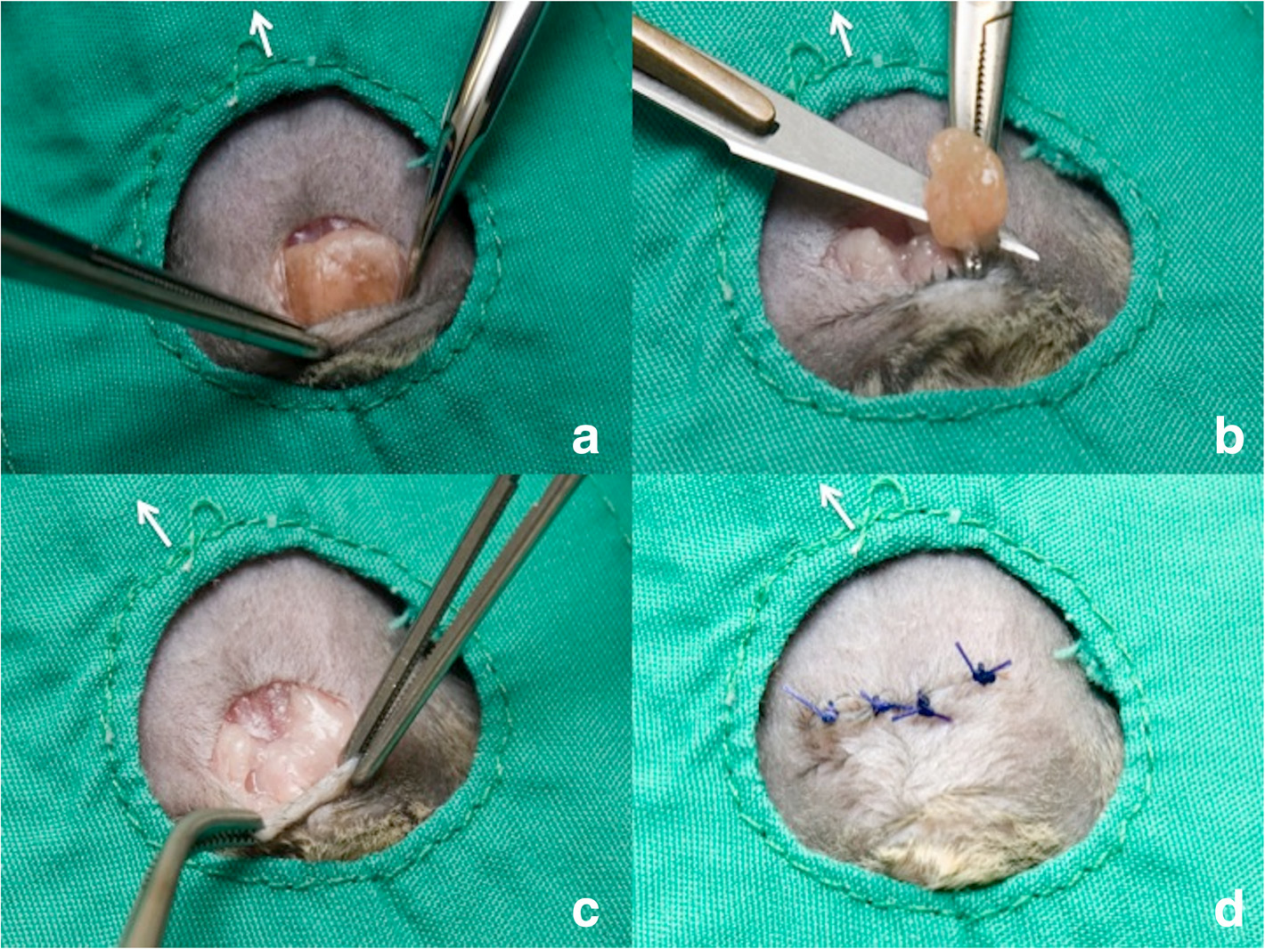
Ablation surgery of preputial glands. **a** Preputial glands exposed (**b**) Gland ablation after haemostat was placed on the duct (**c**) Surgical site after glands are removed (**d**) Interrupted pattern suture to close surgical site. The withe arrow indicates the direction of the head

Anaesthesia was induced with 5 % and maintained with 2.5 % of isoflurane (IsoFlo 100 % w/w Inhalation Vapour, Abbott Laboratories, Maidenhead, UK), in 100 % oxygen. Anaesthesia was maintained for no more than 10 min. Body temperature was maintained with a heat pad set at 38 °C (Homeothermic Blanket system, Harvard Apparatus, Cambridge, UK). Meloxicam (5 mg/g s/c) (Metacam, Boehringer Ingelheim Limited, Bracknell, UK) was administered 10 min prior to surgery for pain relief and enrofloxacin (10 mg/kg s/c)(Baytril 2.5 % solution for injection, Bayer plc, Newbury, UK) as prophylactic antibiotic therapy. The preputial region was prepared for aseptic surgery (http://www.procedureswithcare.org.uk/aseptic-technique-in-rodent-surgery/). A 15 mm transverse skin incision was made cranial to the prepuce using a number 11 scalpel blade. The subcutaneous tissues were gently dissected forward to the prepuce with Metzenbaum scissors and Debakey forceps, without touching the penis. The healthy gland appeared as a flattened circular structure with a cream to light brown colouration (Fig. 1). In contrast the infected gland appeared larger and thin walled with a yellow-red colouration [15]. When a gland was infected, pus was often visible in one lobe of the gland or disseminated throughout. Some glands were embedded in fatty tissue, necessitating its isolation by gentle manipulation with a sterile cotton bud or a swap. Each gland was removed using a hemostat is placed on the ventral side of its duct, followed by cutting proximally with a scalpel blade 11. In animals with abscessation of the gland, adhesions with the skin necessitated very careful dissection before removal. If the gland ruptured during this dissection the surgical wound was thoroughly flushed with sterile saline and an additional dose of enrofloxacin administered at 24 h post-surgery. The skin was closed with polyglactin 910, 4/0 suture (Coated VicrylEthicon, Johnson&Johnson international, Diegrem, Belgium) with an interrupted pattern and skin glue (Indermil xfine, Henkel, Dublin, Ireland) to reinforce the closure (Fig. 2).
